# Overexpressing the *Sedum alfredii* Cu/Zn Superoxide Dismutase Increased Resistance to Oxidative Stress in Transgenic *Arabidopsis*

**DOI:** 10.3389/fpls.2017.01010

**Published:** 2017-06-13

**Authors:** Zhen Li, Xiaojiao Han, Xixi Song, Yunxing Zhang, Jing Jiang, Qiang Han, Mingying Liu, Guirong Qiao, Renying Zhuo

**Affiliations:** ^1^State Key Laboratory of Tree Genetics and Breeding, Chinese Academy of ForestryBeijing, China; ^2^Key Laboratory of Tree Breeding of Zhejiang Province, The Research Institute of Subtropical of Forestry, Chinese Academy of ForestryHangzhou, China

**Keywords:** Cd stress, co-expression network, *Cu/Zn SOD*, oxidative stress, *Sedum alfredii*

## Abstract

Superoxide dismutase (SOD) is a very important reactive oxygen species (ROS)-scavenging enzyme. In this study, the functions of a *Cu/Zn SOD* gene (*SaCu/Zn SOD*), from *Sedum alfredii*, a cadmium (Cd)/zinc/lead co-hyperaccumulator of the Crassulaceae, was characterized. The expression of *SaCu/Zn SOD* was induced by Cd stress. Compared with wild-type (WT) plants, overexpression of *SaCu/Zn SOD* gene in transgenic *Arabidopsis* plants enhanced the antioxidative defense capacity, including SOD and peroxidase activities. Additionally, it reduced the damage associated with the overproduction of hydrogen peroxide (H_2_O_2_) and superoxide radicals (O_2_^•-^). The influence of Cd stress on ion flux across the root surface showed that overexpressing *SaCu/Zn SOD* in transgenic *Arabidopsis* plants has greater Cd uptake capacity existed in roots. A co-expression network based on microarray data showed possible oxidative regulation in *Arabidopsis* after Cd-induced oxidative stress, suggesting that S*aCu/Zn SOD* may participate in this network and enhance ROS-scavenging capability under Cd stress. Taken together, these results suggest that overexpressing *SaCu/Zn SOD* increased oxidative stress resistance in transgenic *Arabidopsis* and provide useful information for understanding the role of *SaCu/Zn SOD* in response to abiotic stress.

## Introduction

Cadmium (Cd), a non-essential trace element, is highly phytotoxic to all living cells through interference with DNA repair machinery, generation of reactive oxygen species (ROS) and disruption of regular gene expression (Valko et al., 2005; Jomova and Valko, 2011). Cd can also result in oxidative stress through interacting with the antioxidative defense system and favoring lipid peroxidation and production of ROS (Rodríguez-Serrano et al., 2006; Woertink et al., 2009).

Plants maintain intracellular ROS levels using a sophisticated antioxidative defense system that includes the antioxidative enzymes superoxide dismutase (SOD), peroxidase (POD), catalase (CAT), peroxiredoxins (Prxs), and glutathione peroxidase (GPX) during their growth and development (Dietz, 2003; Kishikawa and Kuroda, 2014). When exposed to different abiotic stress conditions, plants will increase the accumulation of ROS, leading to enhanced tolerance to oxidative stress by disturbing protein synthesis and stability and damaging cellular macromolecules and membrane lipids in plant cells (Wang et al., 2005). However, plants have the ability to scavenge ROS, allowing control over ROS toxicity and use of ROS as signaling molecules through a complicated gene regulation network (Mittler et al., 2004; Baxter et al., 2014). SOD is one of most important ROS-scavenging enzymes, constituting the first cellular defense against oxidative stress and playing crucial roles in ROS homeostasis due to its conversion of the superoxide anion (O_2_^•-^) to H_2_O_2_ (Gupta et al., 1993; Apel and Hirt, 2004). SODs are classified into three isoforms based on their metal cofactors: manganese (Mn)-SOD, iron (Fe)-SOD and copper (Cu)/zinc (Zn)-SOD. Mn-SOD is primarily localized in mitochondria and peroxisomes; Fe-SOD primarily in chloroplast; Cu/Zn SOD in the cytosol, chloroplasts and peroxisomes (Bueno et al., 1995). Cu/Zn SOD, the most important of the three SOD enzymes, is composed of two subunits combined with Cu and Zn atoms, respectively, that increase the activity and stability of the enzyme (Lin et al., 1995). SODs can alter the O_2_^•-^ to H_2_O_2_ ratio (Chabory et al., 2010), and H_2_O_2_ can modulate the expression of multiple genes, such as transcription factors, antioxidative genes and some stress-related genes. H_2_O_2_ is catalyzed to H_2_O by CAT, Prxs and GPX (Gupta et al., 1993). SODs, along with other enzymes, maintain intracellular ROS homeostasis. SOD overexpression can endow a certain level of tolerance to several stress conditions. For instance, transgenic cassava (*Manihot esculenta*) overexpressing both *MeCu/Zn SOD* and *MeCAT1* showed improved tolerance against oxidative, cold and drought stresses through enhancing ROS-scavenging capability (Xu et al., 2013, 2014). The expression of *PutCu/Zn SOD* was observed in leaves and roots exposed to salt, and oxidative stresses. Transgenic yeast and *Arabidopsis* plants overexpressing *PutCu/Zn SOD* showed increased resistance to multiple abiotic stresses (Wu et al., 2016). In addition, a novel *Cu/Zn SOD* gene from *Saussurea involucrata* Kar. et Kir., *SiCSD*, was induced by cold, drought and oxidative stresses. Overexpression of *SiCSD* in transgenic tobacco improved tolerance to drought, freezing and oxidative stresses (Zhang et al., 2017).

Phytoremediation is considered a gentle, eco-friendly clean-up method. Hyperaccumulator plants can extract metals from the soil and concentrate these metals in their aerial parts. To date, approximately 450 hyperaccumulator species have been identified, but most of them are limited by their biomass and growth rate. *Sedum alfredii* Hance is a new Cd hyperaccumulator that was found inhabiting an abandoned lead (Pb)/Zn mine in southeast China and is an ideal material for investigating the mechanism of heavy metal stress tolerance. Due to its fast growth, the potential application value of *S. alfredii* is tremendous. However, the exact mechanisms that trigger ROS in *S. alfredii* treated with heavy metals are still unclear (Gratão et al., 2005). In our previous study, the transcriptomes from roots, stems and leaves of *S. alfredii* treated with 400 μM CdCl_2_ were identified by high-throughput sequencing (Han et al., 2016), and we found that *SaCu/Zn SOD* expression was highly induced by CdCl_2_ stress. Here, a *SaCu/Zn SOD* gene from *S. alfredii* plants was isolated and functionally characterized. Furthermore, the internal relationships between *SaCu/Zn SOD* and some oxidative genes under Cd stress were explored using a co-expression network. Our results will provide valuable information on the underlying mechanism and possibilities for cultivating forest tree varieties with high resistance level to oxidative stress in the future.

## Materials and Methods

### Plant Materials and Stress Treatments

The Cd-hyperaccumulator plant *S. alfredii* was originally obtained from a Pb/Zn mine in Zhejiang Province, China, cleaned with double-distilled water and grown in a plant growth chamber at 25°C with a 16 h light/8 h dark photoperiod. Thirty-day-old uniform and robust seedlings were treated with 400 μM CdCl_2_ for 0, 0.5, 6, 12, 24, 48, 72 and 96 h. Leaves, roots and stems of seedlings from each sample were harvested at the indicated times, quickly frozen in liquid nitrogen and stored at -80°C for further analyses.

Seeds of *Arabidopsis* were surface-sterilized in 75% (v/v) ethanol for 10 min and then plated on 1/2 Murashige and Skoog (MS) solid medium. One-week-old seedlings were transferred from plates into pots containing a mixture of perlite/soil in a greenhouse at 22°C with a 16 h light/8 h dark photoperiod and 70–75% relative humidity. Four-week-old seedlings were treated with 5 mM CdCl_2_ for 1, 2 and 3 weeks, after which the whole plants were immediately harvested and used for cDNA microarray and physiological analysis.

### Cloning and Analysis of the *SaCu/Zn SOD* Gene

Based on *S. alfredii* transcriptome data, *SaCu/Zn-SOD* was identified and isolated from a cDNA library of *S. alfredii* using the primers SOD-F and SOD-R (**Supplementary Table S1**). The deduced SaCu/Zn-SOD protein sequence and other plant SODs were aligned using CLUSTALX 2.0. A phylogenetic analysis was performed using MEGA 5.2 software with the neighbor-joining method, and the internal branch support was estimated with 1,000 bootstrap replicates.

### Real-time Quantitative RT-PCR (qRT-PCR) Analysis

Total RNA was extracted from *S. alfredii* using a RNA purification kit (NORGEN, Thorold, ON, Canada), and removed genomic DNA contamination. One microgram of total RNA was used for first-strand cDNA synthesis by the Transcriptor First Strand cDNA Synthesis Kit (Roche, Basel, Switzerland). qRT-PCR was performed on a 7300 Real-Time PCR System (Applied Biosystems, United States) using a SYBR PrimeScript^TM^ RT-PCR Kit (TaKaRa, Dalian, China). The PCR program was as follows: 95°C for 10 s, then 40 cycles of 95°C for 5 s and 60°C for 31 s, followed by melting curve analysis. The relative expression levels were calculated according to the method described by Livak and Schmittgen (2001). All the primers are listed in the **Supplementary Table S1**. For each sample, three biological replicates, each with three technical replicates were performed.

### Construction of the Recombinant Plasmid and Plant Transformation

The full-length cDNA of *SaCu/Zn SOD* was cloned into the pBI121G vector driven by the CaMV 35S promoter and introduced into *Arabidopsis* (ecotype Columbia) by the floral dip method (Clough and Bent, 1998). Positive transformants were selected on 1/2 MS solid medium containing 50 mg⋅L^-1^ kanamycin and confirmed by genomic DNA PCR. The expression levels of the transformants were also determined by qRT-PCR. T_3_ homozygous lines were identified by screening for non-segregation from each independent transformant and selected for further biological functional analyses.

### Physiological and Histochemical Staining Analysis

Seeds of wild-type (WT) and T_3_ homozygous overexpression (OE) plants were placed on 1/2 MS agar medium with or without 1.5 mM CdCl_2_ and incubated at 22°C for 3 and 7 days_._ The seed germination rates were determined. Four-week-old seedlings were irrigated with 5 mM CdCl_2_ for 0, 1, 2 and 3 weeks. After the different treatment times, the fresh weight of each plant was calculated.

For the determination of enzyme activities, 1 g leaf samples were harvested from the above Cd-treated plants, homogenized in 8 ml of 50 mM sodium phosphate buffer (PBS, pH 7.8) using a prechilled mortar and pestle, and then centrifuged at 10,000 *g* for 15 min at 4°C. The supernatant was used to measure SOD and POD activities, and O_2_^•-^ levels (Rao et al., 1996). In addition, 0.05 g of leaf tissue was cut into pieces and submerged in a mixture of acetone and ethanol (1:1). The absorbance was measured in a spectrophotometer at 645 and 663 nm to calculate the total chlorophyll contents (Arnon, 1949). Electrolyte leakage was calculated through conductivity measurements (Fan et al., 1997). The malondialdehyde (MDA) and H_2_O_2_ contents were determined according to Velikova et al. (2000).

Rosette leaves harvested from WT and OE *Arabidopsis* plants were immediately soaked in 3,3′ diaminobenzidine (DAB) solution (1 mg⋅mL^-1^, DAB-HCl, pH 3.8) or NBT (1 mg⋅mL^-1^ NBT in 10 mM sodium azide and 10 mM phosphate buffer, pH 7.8), respectively, for 12 h in the dark to detect H_2_O_2_ and O_2_^•-^ contents *in situ*^,^ and then the chlorophyll was eliminated by boiling in 96% ethanol. Images of leaves were acquired using a stereoscopic microscope (LEICA M125, Germany) after chlorophyll elimination. At least three biological replicates were performed for each experiment and approximately 20 leaves harvested from multiple seedlings were inspected.

### Net Cd^2+^ Efflux Measurements

Fifteen-day-old seedlings were treated with 50 μM CdCl_2_ for 24 h and soaked in test liquid [0.1 mM KCl, 0.1 mM CaCl_2_, 0.05 mM CdCl_2_, 0.3 mM 2-(*N*-morpholino) ethanesulfonic acid, pH 5.8] for 15 min. The roots were immobilized on the bottom of a measuring dish in fresh test liquid. The measuring site was 200 μm from the root apex, and the net flux of Cd^2+^ was detected using a non-invasive micro-test technique (NMT; BIO-001A, Younger United States Science and Technology Corp., Beijing, China). The ion flux of Cd^2+^ was calculated according to Fick’s law of diffusion, *J*_0_ *= -D ×* (*d*_C_*/d*_X_), where *J*_0_ is the net ion flux (in μmol⋅cm^-2^ per second), *D* is the self-diffusion coefficient for the ion (in cm^2^⋅s^-1^), *d*_C_ is the difference in the ion concentrations between the two positions, and *d*_X_ is the 10 μm excursion over which the electrode moved in these experiments.

### Microarray and Co-expression Network Construction

Total RNA was isolated from transgenic *Arabidopsis* and WT plants using a RNeasy Plant Mini kit (Qiagen, Hilden, Germany) and purified for microarray data analysis by QuanMai (Shanghai, China). Three OE line constituted a mixed pool as the transgenic group, and three independent biological replicates were carried out. Briefly, double-stranded cDNA was synthesized from 0.2 μg total RNA using a Quick Amp Labeling Kit, One-Color (Agilent). The products were labeled with Cyanine-3-CTP (Cy3) and subsequently hybridized onto the microarray using an Agilent Gene Expression Hybridization Kit. The array was incubated at 65°C for 17 h and then automatically washed and stained using the Gene Expression Wash Pack (Agilent). The probe array was scanned by an Agilent Microarray Scanner (G2505C).

Array images were analyzed to obtain raw data using Feature Extraction software (version10.7.1.1, Agilent Technologies). The data were normalized using the quantile algorithm. Differentially expressed genes were then identified through their fold changes. Gene ontology (GO) was applied to determine the roles of these differentially expressed genes. Based on the microarray data, a co-expression network was constructed using the R package WGCNA^1^ (Langfelder and Horvath, 2008). All the parameters were set at the default values except the default power, which was set to six (R Core Team, 2014). The outputted, co-expressed genes having strong interconnections were defined as hub genes. We then chose specific genes from the hub genes for further analyses with FunNet^2^ and AmiGO 2^3^. The co-expression network was constructed using Cytoscape software (Saito et al., 2012).

### Statistical Analysis

At least 10 plants were analyzed in each treatment. All the experiments were repeated at least three times. The results are shown as the means ± SD. Statistical determinations were carried out using a one-way ANOVA test, and differences between two groups of data for comparisons in all the experiments were evaluated as statistically significant (^∗^*p* < 0.05) or extremely significant (^∗∗^*p* < 0.01).

## Results

### Isolation and Sequence Analysis of *SaCu/Zn SOD*

**FIGURE 1:**
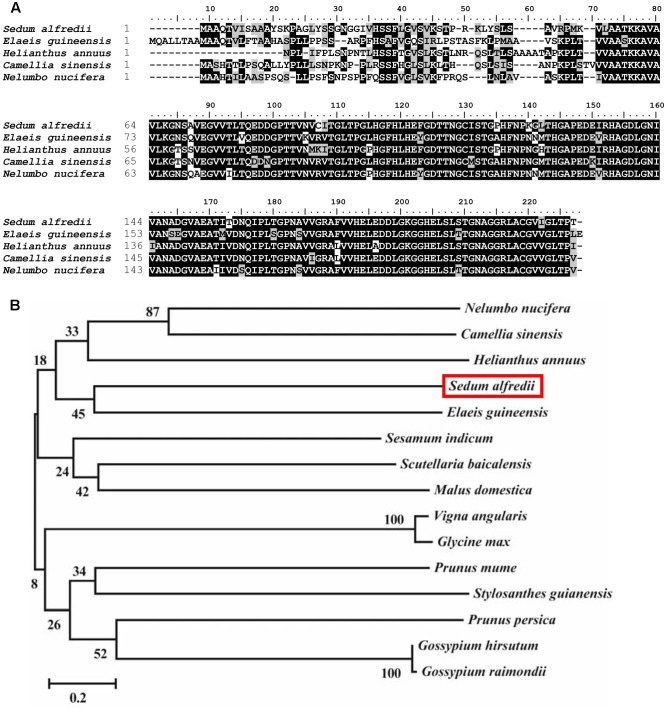
Comparison of amino acid sequences of SaCu/Zn SOD and closely related Cu/Zn SODs from other plants. **(A)** Alignment of the amino acid sequence of SaCu/Zn SOD with Cu/Zn SOD proteins from different plants species. **(B)** The phylogenetic relationships between SaCu/Zn SOD and other plant Cu/Zn SOD proteins. The neighbor-joining phylogenetic tree was created with ClustalW using MEGA 5.2. All amino acid sequences were retrieved from GenBank: *S. alfredii* Cu/Zn SOD (AII25435.1); *Elaeis guineensis* Cu/Zn SOD (XP_010940533.1); *Helianthus annuus* Cu/Zn SOD (CAH06449.1); *Camellia sinensis* Cu/Zn SOD (AKN10571.1); *Nelumbo nucifera* Cu/Zn SOD (XP_010267263.2); *Scutellaria baicalensis* Cu/Zn SOD (AEO27875.1); *Prunus persica* Cu/Zn SOD (AFG19614.1); *Vigna angularis* Cu/Zn SOD (XP_017433773.1); *Sesamum indicum* Cu/Zn SOD (XP_011088797.1); *Glycine max* Cu/Zn SOD (XP_003538169.1); *Gossypium hirsutum* Cu/Zn SOD (XP_016671933.1); *G. raimondii* Cu/Zn SOD (XP_012485000.1); *P. mume* Cu/Zn SOD (XP_008226630.1); *Stylosanthes guianensis* Cu/Zn SOD (AKO62676.1); *Malus domestica* Cu/Zn SOD (XP_008365087.1).

The full-length cDNA of *SaCu/Zn SOD* (KF806556) is 1,001 bp in length, encodes a 210 amino acid protein with a predicted molecular weight of 39.1 kDa and an isoelectric point of 6.41, which is flanked by stretches of 48 and 53 bp at the 5′- and 3′-untranslated regions, respectively. A multiple sequence alignment with Cu/Zn SODs from other plants was carried out and showed that the SaCu/Zn SOD protein exhibited the same familial signature, including a highly conserved active site (**Figure 1A**). The evolutionary relationships among SaCu/Zn SOD and other plant Cu/Zn SODs showed that SaCu/Zn SOD was most similar to the Cu/Zn SOD from *Prunus persica* and had a long evolutionary distance from the Cu/Zn SODs of *P. trichocarpa* (**Figure 1B**). We further investigated the genomic structure of *SaCu/Zn SOD* and found that *SaCu/Zn SOD* was approximately 1,985 bp, containing seven introns and eight exons, which is similar to the characteristics of the gene structures in *P. trichocarpa* and *Arabidopsis* (**Supplementary Figure S1**).

### Expression Patterns of *SaCu/Zn SOD* in Different Tissues and under Cd Stress

**FIGURE 2:**
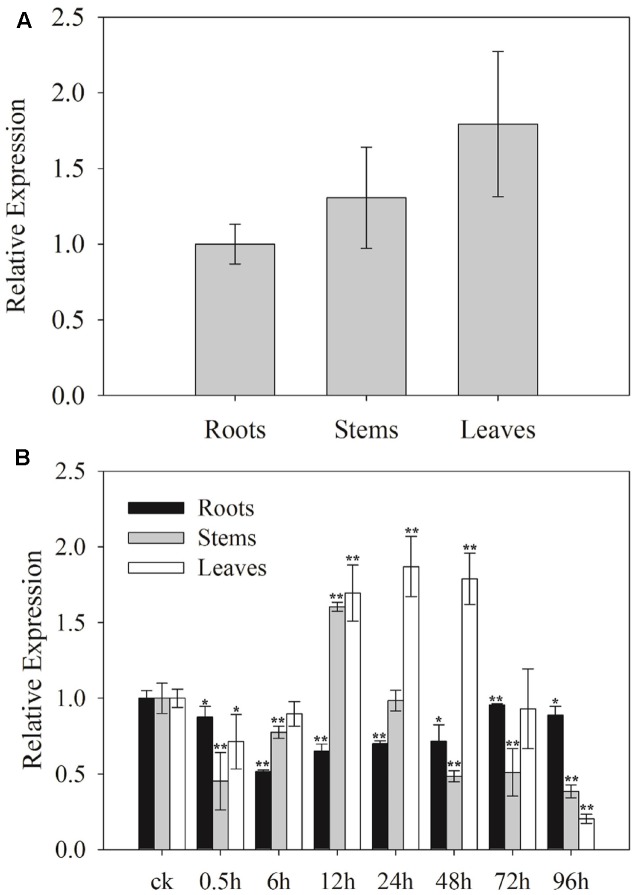
Expression profiles of *SaCu/Zn SOD* in different tissues under CdCl_2_ stress. **(A)** Tissue-specific expression of *SaCu/Zn SOD* by qRT-PCR. Total RNA was extracted from the roots, stems and leaves of 25-day-old seedlings. **(B)** Expression levels of *SaCu/Zn SOD* in the roots, stems and leaves under CdCl_2_ stress. The 25-day-old seedlings were obtained by hydroponic culturing with 400 μM CdCl_2_ for the indicated times. For each sample, three technical replicates were performed. The error bars were calculated from the multiple replicates of qRT-PCR. Bars indicate means ± SD (*n* = 10). Asterisks indicate significant differences at ^∗^*p* < 0.05 and ^∗∗^*p* < 0.01 based on a *t*-test.

To determine the potential functions of *SaCu/Zn SOD*, the relative expression levels of *SaCu/Zn SOD* in roots, stems and leaves of *S. alfredii* seedlings were tested using qRT-PCR. The expression levels of *SaCu/Zn SOD* in the leaves, stems and roots were different. The *SaCu/Zn SOD* was expressed at a higher level in the leaves and at a lower level in the roots (**Figure 2A**). To further determine the effects of Cd stress on the expression of *SaCu/Zn SOD*, 30-day-old seedlings were exposed to 400 μM CdCl_2_ for different time periods. The expression of *SaCu/Zn SOD* was initially reduced, then gradually increased and peaked at 12 h of stress in the stems and 24 h in the leaves, respectively. The *SaCu/Zn SOD* expression did not change significantly in the roots during CdCl_2_ stress (**Figure 2B**).

### Plant Growth and Cd Uptake under Different Cd Treatments

**FIGURE 3:**
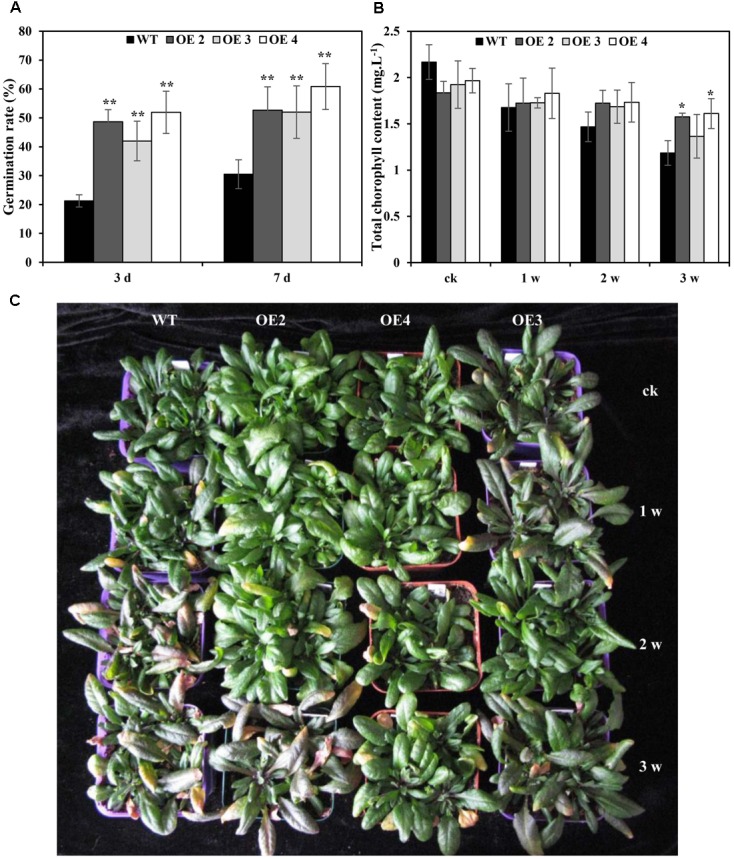
Overexpression of *SaCu/Zn SOD* in transgenic plants enhanced Cd tolerance. **(A)** Germination rates of *SaCu/Zn SOD* transgenic (OE2, OE3, and OE4) and WT plants on 1/2 MS agar medium supplemented with 1.5 mM CdCl_2_. Germination rates were determined after incubation at 22°C for 3 and 7 days. **(B,C)** Comparison of the chlorophyll contents and phenotypes between *SaCu/Zn SOD* transgenic and WT *Arabidopsis* plants. Four-week old seedlings were irrigated with 5 mM CdCl_2_ for 0, 1, 2 and 3 weeks, and the photographs of seedlings were taken. The error bars represent the standard deviations of the average values. Asterisks indicate significant differences at ^∗^*p* < 0.05 and ^∗∗^*p* < 0.01.

To further determine the effects of *SaCu/Zn SOD* under Cd stress, seven T_3_ homozygous transgenic *Arabidopsis* plants constitutively expressing *SaCu/Zn SOD* were generated and confirmed by genomic DNA PCR and qRT-PCR (**Supplementary Figure S2**). Three representative transgenic lines (OE2, OE3, and OE4) were selected for further research. There was no difference in the seed germination rate between transgenic and WT *Arabidopsis* plants without CdCl_2_ treatment, but transgenic lines showed higher germination rates than WT plants under CdCl_2_ stress (**Figure 3A** and **Supplementary Figure S3**). After 1 week of Cd treatment, WT plants showed visual Cd toxicity symptoms, such as wilting and etiolation. The toxicity symptoms became more severe with increasing exposure time, but the toxicity symptoms in transgenic *Arabidopsis* were less severe than those of WT during the same exposure time (**Figure 3C**). Meanwhile, the chlorophyll contents decreased in transgenic and WT plants under Cd stress, but all OE lines had higher contents than WT plants (**Figure 3B**). Additionally, the fresh weights of transgenic *Arabidopsis* decreased by 10.2, and 14.0% under 2 and 3 weeks of treatment, respectively; however, greater decreases of 13.7 and 19.6%, respectively, were observed in WT (**Supplementary Figure S4**).

**FIGURE 4:**
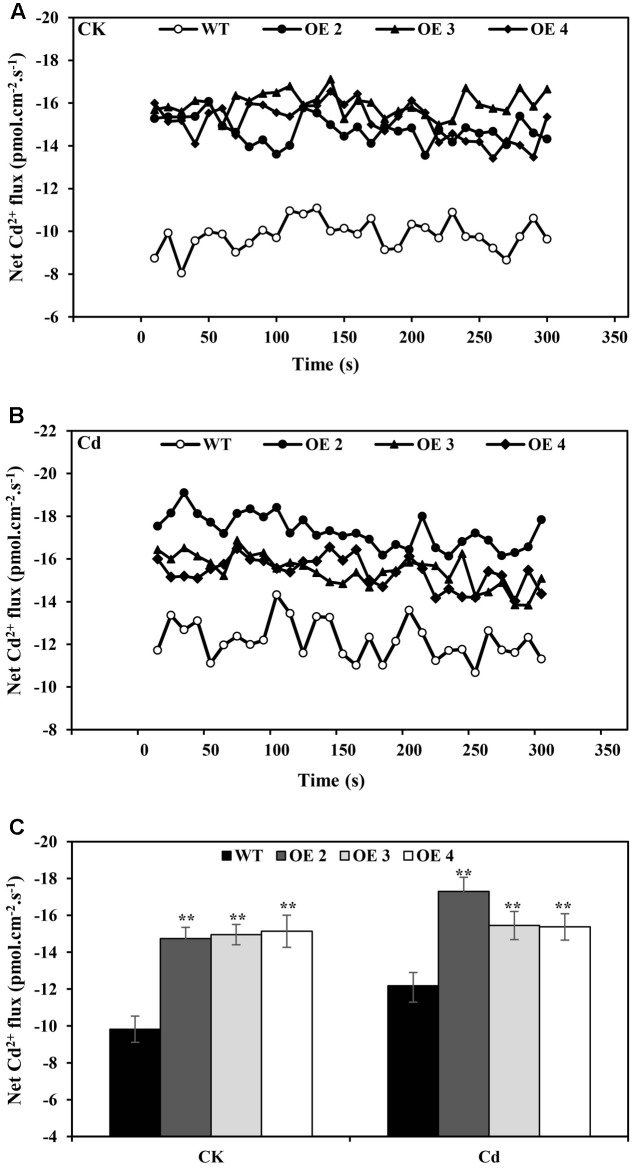
Net Cd^2+^ fluxes. Net Cd^2+^ fluxes in the roots of transgenic (OE2, OE3, and OE4) and WT plants treated without **(A)** or with CdCl_2_ stress **(B)**, respectively. The average 300 s net Cd^2+^ fluxes **(C)** are illustrated to highlight the trend differences. Bars indicate means ± SD. Asterisks indicate significant differences at ^∗^*p* < 0.05 and ^∗∗^*p* < 0.01.

A non-invasive micro-test (NMT) technique was used to investigate the Cd^2+^ uptake in root tips of OE and WT plants. Under normal conditions, the net Cd^2+^ fluxes of OE and WT were within a relatively small range, but the Cd^2+^ influxes of OE lines were slightly higher than those of WT plants. With 50 μM CdCl_2_ supplementation, the net Cd^2+^ flux was affected (**Figure 4**), while OE lines exhibited higher influx than did WT plants. These results indicated that a greater Cd uptake capacity existed in these roots than in WT plants.

### *SaCu/Zn SOD* Participates in ROS Accumulation and Scavenging

**FIGURE 5:**
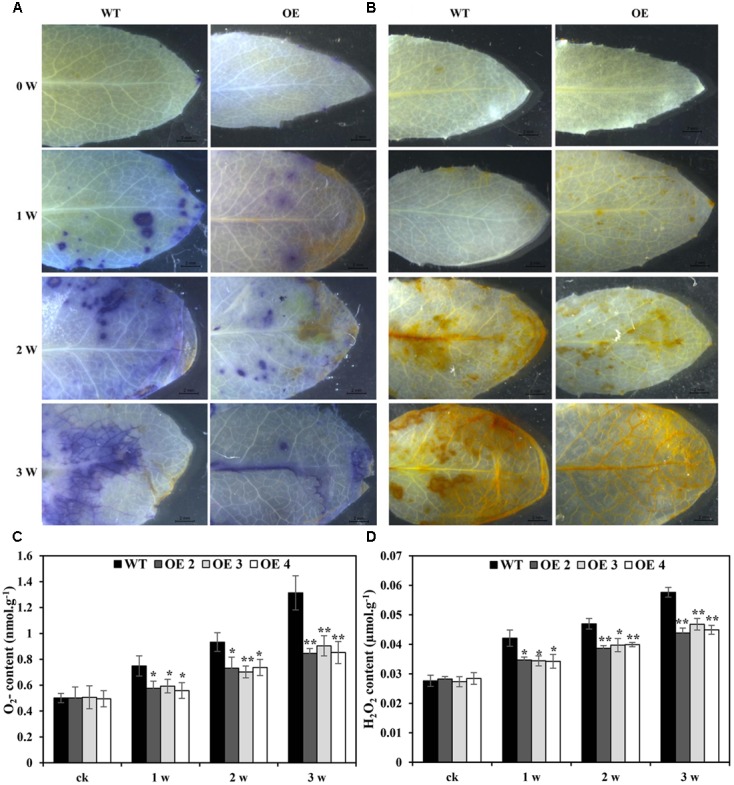
Comparison of Cd-induced O_2_^-^ and H_2_O_2_ levels between WT and transgenic *Arabidopsis* plants. Four-week old seedlings were irrigated with 5 mM CdCl_2_ for 0, 1, 2, and 3 weeks. Leaves were detached from transgenic (OE2, OE3, and OE4) and WT *Arabidopsis* plants and the O_2_^-^ and H_2_O_2_ levels were visualized by NBT **(A)** and DAB staining **(B)**. **(C,D)** Comparison of the O_2_^-^ and H_2_O_2_ contents between WT and transgenic *Arabidopsis* plants. Bars indicate means ± SE. Asterisks indicate significant differences at ^∗^*p* < 0.05 and ^∗∗^*p* < 0.01.

**FIGURE 6:**
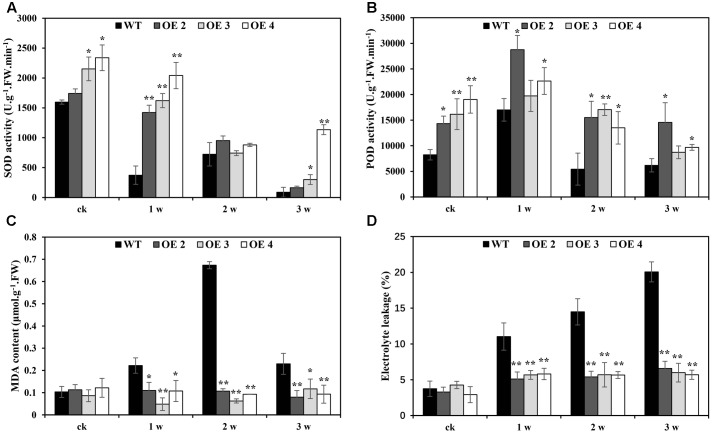
Physiological analysis of WT and *SaCu/Zn SOD* transgenic *Arabidopsis* plants. Four-week old seedlings were irrigated with 5 mM CdCl_2_ for 0, 1, 2, and 3 weeks and the leaves were collected to measure SOD **(A)** and POD **(B)** activities, MDA contents **(C)** and electrolyte leakage **(D)**. Bars indicate means ± SE (*n* = 20–25 plants for each genotype). Asterisks indicate significant differences at *^∗^p* < 0.05 and ^∗∗^*p* < 0.01.

The effects of Cd toxicity on the production of ROS were analyzed by NBT and DAB staining, which produced dark blue and deep brown products, respectively. Both NBT and DAB staining showed that the O_2_^•-^ and H_2_O_2_ levels in transgenic and WT plants exhibited no differences under normal growth conditions. However, under Cd stress conditions, the accumulation of O_2_^•-^ and H_2_O_2_ in transgenic lines was obviously lower than that in WT plants (**Figures 5A,B**). Additionally, compared with WT plants, the O_2_^•-^ and H_2_O_2_ contents in transgenic *Arabidopsis* plants overexpressing *SaCu/Zn SOD* were lower under Cd stress condition (**Figures 5C,D**). We next tested the activities of SOD and POD. After Cd treatment, these enzyme activities in the OE lines were significantly greater than those in WT plants (**Figures 6A,B**). This indicated that overexpression of *SaCu/Zn SOD* in *Arabidopsis* might enhance the ability to scavenge ROS. The degrees of oxidative damage in the leaves were determined by testing MDA levels and electrolyte leakage. Transgenic and WT plants showed similar growth levels without Cd stress. However, MDA levels and electrolyte leakage in the transgenic plants were significantly lower than those in WT plants under Cd stress (**Figures 6C,D**). These results suggested that *SaCu/Zn SOD* confers Cd stress tolerance through increasing the ability of ROS-scavenging and reducing membrane injury.

### Expression Analysis of Antioxidant-related Genes in *SaCn/Zn SOD*-overexpressing Plants

**FIGURE 7:**
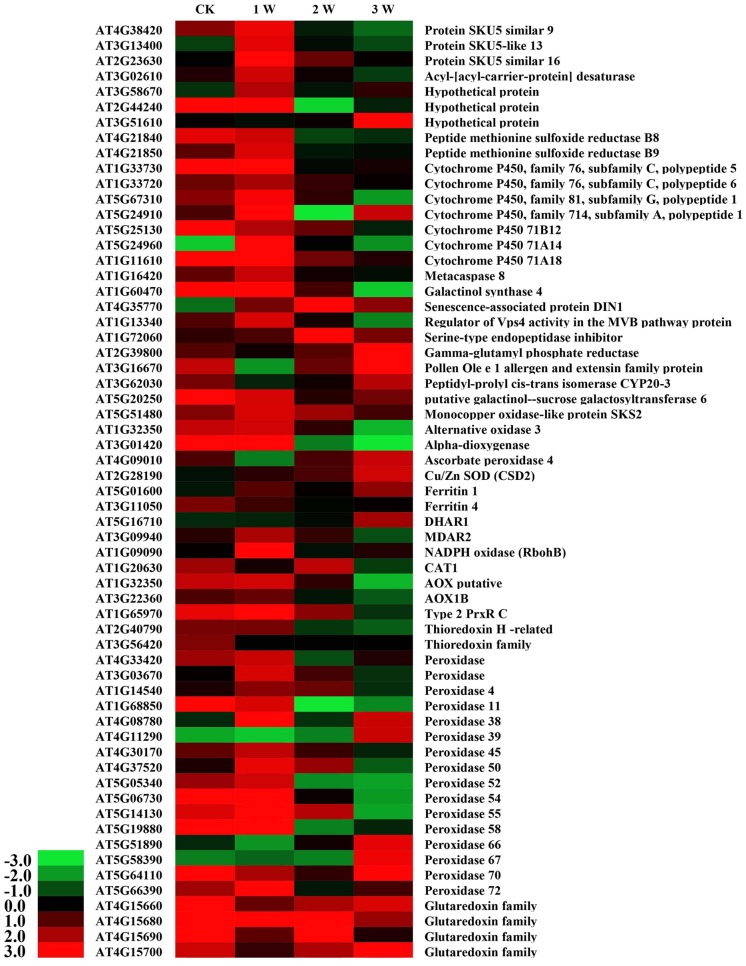
DEG expression profiles in *Arabidopsis* under CdCl_2_ stress at different time points. All the ratios are log2 transformed so that inductions and repressions of identical magnitude are numerically equal but opposite in sign. Log ratios of 0 (ratios of 1) are colored black, and increasingly positive (induction) or negative (repression) log ratios are colored red or green, respectively, with increasing intensity.

Cadmium stress rapidly induced a marked increase in ROS and altered the antioxidant contents in WT and OE plants. To further investigate the transcriptional regulatory profiles of *SaCn/Zn SOD*, an Agilent *Arabidopsis* Oligo Microarray assay was employed. The microarray data showed that 2,966–6,030 genes were involved in this process during different treatment durations (**Supplementary Table S2**). Based on the microarray data, GO classification was conducted for the pathways significantly (*p* < 0.05) enriched in the genes that were differentially regulated under Cd stress. Numerous genes with known function were classified into the GO terms: “Biological process,” “Cellular component,” and “Molecular function” (**Supplementary Figure S5**). Among these, 61 cadmium stress-responsive genes involved in metal transport, binding to the cell wall, metal chelation in the cytosol, repair of stress-damaged proteins and antioxidative enzyme activity were selected to illustrate the molecular/cellular mechanisms in plants (Yang et al., 2005). As shown in **Figure 7**, these 61 genes were differentially up- and down-regulated under different treatment time points (**Figure 7**). Four members of the glutaredoxin family were up-regulated to different levels at the different time points. Furthermore, 16 peroxidase genes were found in the microarray data. Five of them [*peroxidase 38* (AT4G08780), *peroxidase 39* (AT4G11290), *peroxidase 66* (AT5G51890), *peroxidase 67* (AT5G58390), and *peroxidase 70* (AT5G64110)] were significantly up-regulated in the 3rd week. By contrast, another five peroxidase genes [*peroxidase 11* (AT1G68850), *peroxidase 50* (AT4G37520), *peroxidase 52* (AT5G05340), *peroxidase 54* (AT5G06730), and *peroxidase 55* (AT5G14130)] were down-regulated in the 3rd week. In addition to these two gene families, two ferritin genes [*ferritin 1* (AT5G01600) and *ferritin 4* (AT3G11050)], one catalase gene [*CAT1* (AT1G20630)] and one thioredoxin-dependent peroxidase gene [Type 2 *PrxR C* (AT1G65970)] showed higher expression levels. The genes involved in these pathways were highly activated, indicating that these pathways are important for cadmium stress response.

### Co-expression Network Construction

**FIGURE 8:**
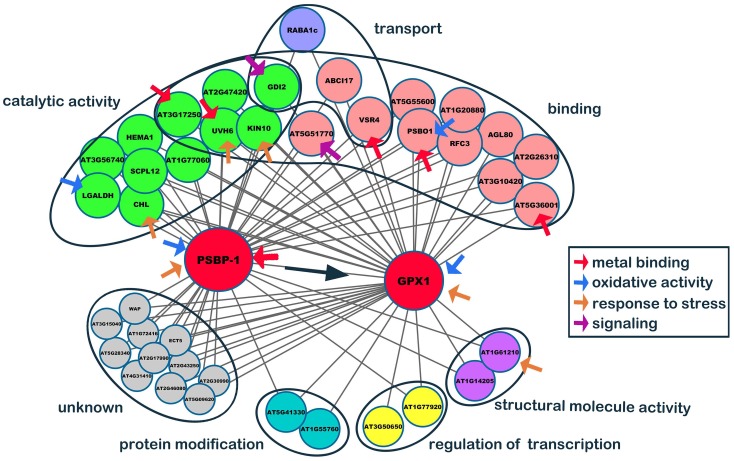
Co-expression network of *PSBP2-1* and *GPX1*. From the top 50 hub genes, two major genes, oxygen-evolving enhancer protein 2-1 (*PSBP2-1*) and phospholipid hydroperoxide glutathione peroxidase 1 (*GPX1*), which might directly relate to oxidative stress, were selected, and a stress-response co-expression subnetwork was constructed by the Cytoscape software.

To determine the genes affected by *SaCu/Zn SOD* in *Arabidopsis*, we constructed a co-expression network based on the normalized microarray data. After excluding non- and lowly expressed genes, we identified 45 gene co-expression modules. Ultimately, the resulting network was consisted of 21 distinct gene modules, and then the top 50 hub genes were selected based on coefficients of variance (**Supplementary Table S3**). From the top 50 hub genes, two major genes, oxygen-evolving enhancer protein 2–1 (*PSBP-1*, A_84_P20343, AT1G06680) and phospholipid hydroperoxide *GPX1* (A_84_P815195, AT2G25080), that might be directly relate to oxidative stress (**Supplementary Table S4**) were selected to constructed a stress-response co-expression subnetwork. As shown in **Figure 8**, *PSBP-1* was regarded as the key co-expression gene, being related to 95% of edges and another hub gene, *GPX1* (At1G55760 and At3G50650 interacted with *GPX1* only). The functions of 11 edges (unknown genes) were not clear, and the other edges were classified into 6 categories. Of the 40 edges, 11 (∼27.5%) had catalytic activities, while 16 (∼40.0%) had binding functions. The binding targets were nucleotides, chromatins, fatty acids, metal ions, adenosine triphosphate and nicotinamide adenine dinucleotide phosphate. Red arrows indicated the metal binding genes, and *PSBP-1*, *PSBO1*, and *VSR4* interacted with calcium ions. AT5G36001 interacted with zinc ions, while AT3G17250 bound to Mn^2+^/Mg^2+^ for further processing. *PSBO1*, *LGALDH* and these two hub genes were oxidative stress-related genes (blue arrows). *GDI2* and AT5G51770 (purple arrows) participated in signal transduction. In addition, we classified six stress-response genes (yellow arrows), which were involved in catalysis, metal binding, and structural integrity.

## Discussion

### Effect of Cd Stress on *SaCu/Zn SOD* Expression

Cadmium is known to generate and lead to accumulation of ROS in plants, resulting in accelerated cell death (Foyer and Noctor, 2005). At moderate levels, ROS play an important role as signal molecules in the regulation of numerous biological processes (Baxter et al., 2014). Cd concentrations in the leaves and stems of *S. alfredii* reached maximums of 9,000 and 6,500 mg/kg, respectively, at a treatment level of 400 μM Cd (Yang et al., 2004). These values indicated that aerial plant parts could accumulate Cd and suffer from severely Cd stress. Initially, Cd^2+^ is readily taken up by the roots and translocated into stems and leaves. Then, ROS, as a molecular signal, cause toxicity in plants. In this study, the *SaCu/Zn SOD* expression initially decreased after 0.5 and 6 h of Cd treatment. However, to protect stem and leaf tissues from ROS damage, its expression level gradually increased and finally reached a peak (12 and 24 h).

### Effect of Cd Stress on Growth, Oxidation Resistance, and Cd Uptake

Cadmium can enter plant cells, producing oxidative stress, inhibiting chlorophyll synthesis and disturbing photosynthetic metabolism, destroying membrane integrity, decreasing nutrient uptake and inhibiting plant growth (Schützendübel et al., 2001; Pietrini et al., 2003). In this study, the etiolation of WT was severe due to Cd exposure, and the chlorophyll content confirmed this phenomenon. However, transgenic *Arabidopsis* plants overexpressing *SaCu/Zn SOD* showed improved seed germination and increased plant growth under Cd stress conditions (**Figure 3** and **Supplementary Figure S4**), indicating that Cd inhibited plant growth through inhibiting of chlorophyll synthesis and impairing photosynthesis. Consistently, overexpression of pea chloroplast *Cu/Zn-SOD* in tobacco improved photosynthetic performance under moderate stress and maintained the photosynthetic capacity after severe oxidative stress (Gupta et al., 1993).

Plants possess very efficient enzymatic antioxidant defense systems, which work in concert to control cascades of excessive oxidation and protect plant cells from oxidative damage by scavenging ROS (Gill and Tuteja, 2010). In the present study, the enhanced SOD and POD enzyme activities, decreased damage to membrane integrity, and lowered O_2_^•-^ and H_2_O_2_ contents in transgenic *Arabidopsis* under Cd stress (**Figures 5**, **6**) indicating that these plants were more resistant to oxidation than WT plants. We hypothesize that transgenic tissues have lower O_2_^•-^ and H_2_O_2_ levels, leading to higher net rates of photosynthesis and reduced oxidative damage under stressful conditions. The protective effects of overexpression of *SaCu/Zn SOD* in *Arabidopsis* plants may involve two strategies: enhancing ROS scavenge efficiency and net rates of photosynthesis and/or decreasing damage caused by lipid peroxidation and damage affecting plasma membrane integrity.

Cadmium uptake and plasma membrane integrity have internal correlations in transgenic and WT plants (**Figures 4**–**6**). Histochemical staining and the quantification of the loss of plasma membrane integrity suggested that Cd exposure increased the levels of membrane damage in both WT and transgenic plants. It would be interesting to analyze results showing that the Cd^2+^ influx of OE lines increased under normal growth conditions or Cd stress, but the Cd accumulation of transgenic and WT plants was not significantly different (data not shown). Mühling and Läuchli (2003) reported that disturbed membrane functions may cause elevated Cd concentrations. Cd stress may damage plasma membrane integrity, resulting in more Cd^2+^ being transported into the cell and accumulating in WT plants. The Cd^2+^ flux increased, implying the possibility of more Cd accumulation in OE lines; then at last the Cd contents of WT and OE were similar. The Cd accumulation due to cell damage influenced the state of WT plants, but the effects of *SaCu/Zn SOD* overexpression in transgenic *Arabidopsis* decreased the damage.

### Regulatory Network under Cd Stress

Several reports have indicated that a high antioxidative capacity is required for heavy metal hyperaccumulation in plants (Cho and Seo, 2005; Wang et al., 2008). The antioxidative system is composed of non-enzymatic (ascorbate, glutathione, α-tocopherol, and carotenoid) and ROS-scavenging enzymes (SOD, POD, CAT, ascorbate peroxidase) (Mittler et al., 2004). In this study, transgenic *Arabidopsis* accumulated less H_2_O_2_ and O_2_^•-^ than WT plants (**Figure 5**), which suggested that the former have a high ROS-scavenging capacity. According to microarray analysis, the most highly expressed antioxidant-related genes were the member of glutaredoxin and peroxidase family (**Figure 7**). In addition to prominent antioxidant enzymes, we also found many other enzymes in the microarray data, such as alternative oxidase (AOX), dioxygenases, and peptidyl proyl *cis–trans* isomerases (PPIase). AOX is a terminal oxidases catalyzing the reduction of oxygen to water in the plant mitochondrial electron transport chain (Millar et al., 2011). The ability of AOX to maintain critical mitochondrial and chloroplastic functions during extreme drought is likely due to its ability to reduce oxidative damage (Dahal and Vanlerberghe, 2017). Dioxygenases are non-heme iron-containing enzymes important in the biosynthesis of plant signaling compounds such as abscisic acid, gibberellins, and ethylene and of secondary metabolites, notably flavonoids and alkaloids (Prescott and John, 1996). Overexpression of a dioxygenase gene in *Nicotiana plumbaginifolia* increases abscisic acid and phaseic acid levels and enhances drought tolerance (Qin and Zeevaart, 2002). PPIases are ubiquitous proteins that are found in the cytosols of both prokaryotic and eukaryotic cells, and have proposed functions in protein folding, protein degradation, stress response and signal transduction (Laxa et al., 2007). In this study, a gene similar to *Arabidopsis AOX3* was significantly down-regulated at the 3rd week, but the expression levels of dioxygenase and PPIase were not sharply changed. We speculate that overexpression of *SaCu/Zn SOD* gene may mainly affect the high expression of antioxidative-related genes such as glutaredoxin and peroxidase, which interact with Cu/Zn SOD in *Arabidopsis* and work together to scavenge ROS, rather than AOX, dioxygenases and PPIase.

The co-expression network may reveal a possible model of *SaCu/Zn SOD* action in transgenic *Arabidopsis* after Cd-induced oxidative stress. Cd can affect many vital processes, including a series of genes, especially stress-related genes, interact with oxidative genes under Cd toxicity. Subsequently, catalysis and binding play important roles in Cd-exposed *Arabidopsis*, and many metal ions participate in these activities. Iron-sulfur (Fe-S) cluster proteins, such as UVH6, with Fe as a cofactor are essential to photosynthesis and oxidation-reduction reactions (Zhang, 2015). Zn^2+^ is also a cofactor for many enzymes, such as AT5G36001, which plays an essential role in transcription of many antioxidant genes (Hansen et al., 2007). The binding of Ca^2+^, Fe^2+^, Mn^2+^ and Mg^2+^ is also closely related to Cd response (Groten et al., 1991). The genetic and physiological processes of plants are strongly associated due to genes having two or more simultaneous functions at the same time, and we hypothesize that the overexpression of *SaCu/Zn SOD* may participate in this co-expression network against Cd-induced oxidative stress in some unclear way.

## Conclusion

**FIGURE 9:**
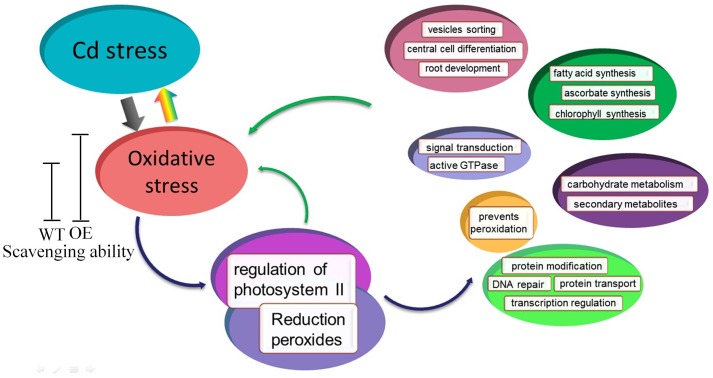
Model of *SaCu/Zn SOD* mediating Cd stress response in transgenic *Arabidopsis*. Transgenic *Arabidopsis* overexpressing *SaCu/Zn SOD* exhibit increased the expression of *SaCu/Zn SOD*, and as a result, these plants have greater ROS scavenging ability than WT plants. Under Cd stress, plants suffer from oxidative stress and then regulate the photosystems and peroxides. The stress response involves six categories of biological process such as signal transport, material synthesis, metabolism, protein modification, DNA repair, etc.

Based on the microarray data and co-expression network, we developed a schematic model for transgenic *Arabidopsis* with enhanced oxidation under Cd stress (**Figure 9**). Cd stress induces the production of ROS, which are highly reactive molecules, leading to oxidative stress. Under normal conditions, the lower levels of ROS in WT and OE plants play important roles as signaling molecules in numerous biological processes. However, Cd stress induces ROS overproduction and oxidative stress. Transgenic *Arabidopsis* plants have a greater ability to scavenge ROS compared with WT plants, so they have better growth than WT plants. Additionally, the expression and regulation of photosystem II and reduction peroxides were mediated by oxidative stress induced by Cd stress. Thus, *SaCu/Zn SOD* in *Arabidopsis* might act on the two hub genes, and together these genes regulate central cell differentiation, signal transduction, protein modification and secondary metabolites.

In summary, *SaCu/Zn SOD* may be responsible for conferring Cd tolerance. Additional modifications of other active-oxygen-scavenging system components in plants will help to elucidate the interactions among these protective mechanisms. We remain hopeful that investigations of this type will eventually provide significant improvements in the tolerance of cultivated plants to Cd stress.

## Author Contributions

RZ and ZL conceived and designed the experiments. ZL, XS, and XH performed the experiments. JJ, QH, YZ, ML, and GQ analyzed the data. ZL, XH, and XS wrote the manuscript. All of the authors read and approved the final manuscript.

## Conflict of Interest Statement

The authors declare that the research was conducted in the absence of any commercial or financial relationships that could be construed as a potential conflict of interest.

## Footnotes

1 http://labs.genetics.ucla.edu/horvath/Co-expressionNetwork/Rpackages/WGCNA/Tutorials/index.html

2 http://www.funnet.ws

3 http://amigo.geneontology.org/amigo/landing

## Supplementary Material

FIGURE S1 Comparison of the genomic DNA sequences of *SaCu/Zn SOD* and other plant *Cu/Zn SOD* genes.

FIGURE S2 PCR detection of transgenic *Arabidopsis*. **(A)** *SaCu/Zn SOD* expression levels in seven independent lines of *SaCu/Zn SOD* transgenic and WT *Arabidopsis* plants. **(B,C)** PCR analysis of *SaCu/Zn SOD* -transformed *Arabidopsis* using vector primers **(B)** and gene primers **(C)**. M: DNA Marker DL2000; 1–2: The WT line and water were used as negative control; 3–10: *SaCu/Zn SOD*-transformed *Arabidopsis* plant lines; 11: The recombinant plasmid pBI121G-SaCu/Zn SOD.

FIGURE S3 Germination status of transgenic *Arabidopsis* seeds after 7 days on a culture dish. The seeds of *SaCu/Zn SOD* transgenic (OE2, OE3, and OE4) and WT plants were surface-sterilized and plated on 1/2 MS agar medium plates supplemented with 1.5 mM CdCl_2_ and grown 7 days.

FIGURE S4 Fresh weights of transgenic and WT *Arabidopsis* exposed to Cd for different time periods. Bars indicate means ± SD. Asterisks indicate significant differences at *^∗^p* < 0.05 and ^∗∗^*p* < 0.01.

FIGURE S5 Gene ontology analyses of the differentially regulated genes identified by microarray analysis under normal growth and Cd stress conditions. **(A–D)** indicate 0 week (ck), 1 week, 2 weeks, and 3 weeks, respectively. GO analysis of each period includes “Biological process,” “Cellular component,” and “Molecular function”.

TABLE S1 Primers used in this study.

TABLE S2 Differentially expressed genes that are up- and down-regulated at least 2-fold in *SaCn/Zn SOD* transgenic *Arabidopsis* compared with WT plants under CdCl_2_ stress.

TABLE S3 A complete list of top 50 hub gene module assignments.

TABLE S4 Edge genes annotated in the co-expression subnetwork of *PSBP-1* and *GPX1*.
