# Supplementary figures and images for: Overexpressing the Sedum alfredii Cu/Zn Superoxide Dismutase Increased Resistance to Oxidative Stress in Transgenic Arabidopsis
Source: Front Plant Sci. 2017 Jun 13;8:1010. doi: 10.3389/fpls.2017.01010 (PMC5469215; doi:10.3389/fpls.2017.01010)

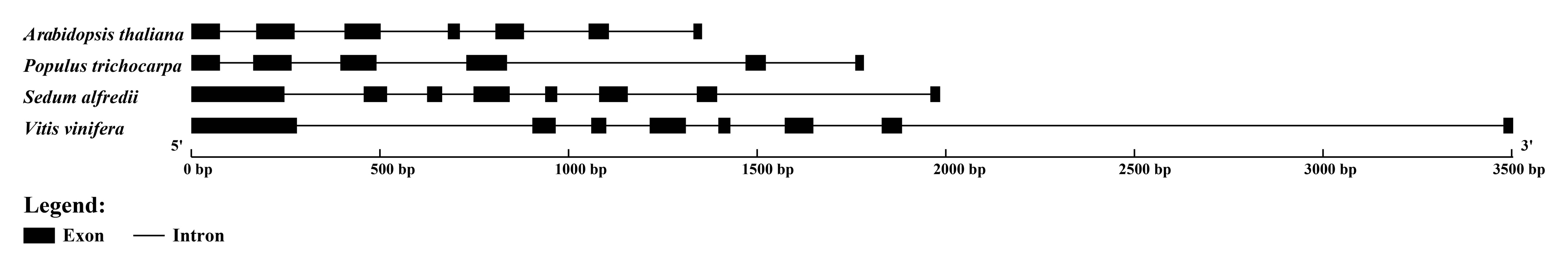

Supplement: FIGURE S1 — Comparison of the genomic DNA sequences of SaCu/Zn SOD and other plant Cu/Zn SOD genes. [file Image_1.JPEG]

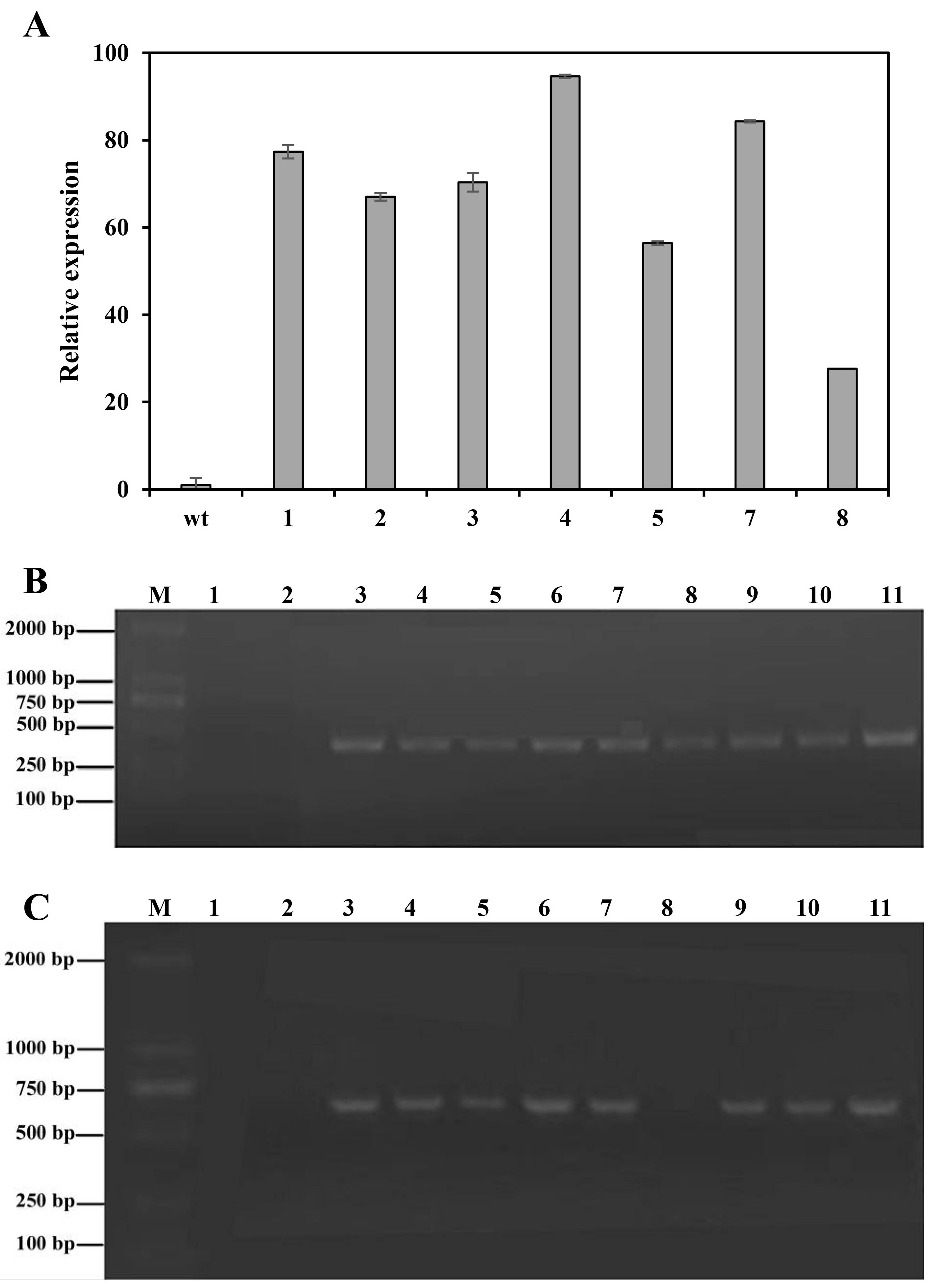

Supplement: FIGURE S2 — PCR detection of transgenic Arabidopsis. (A) SaCu/Zn SOD expression levels in seven independent lines of SaCu/Zn SOD transgenic and WT Arabidopsis plants. (B,C) PCR analysis of SaCu/Zn SOD -transformed Arabidopsis using vector primers (B) and gene primers (C). M: DNA Marker DL2000; 1–2: The WT line and water were used as negative control; 3–10: SaCu/Zn SOD-transformed Arabidopsis plant lines; 11: The recombinant plasmid pBI121G-SaCu/Zn SOD. [file Image_2.JPEG]

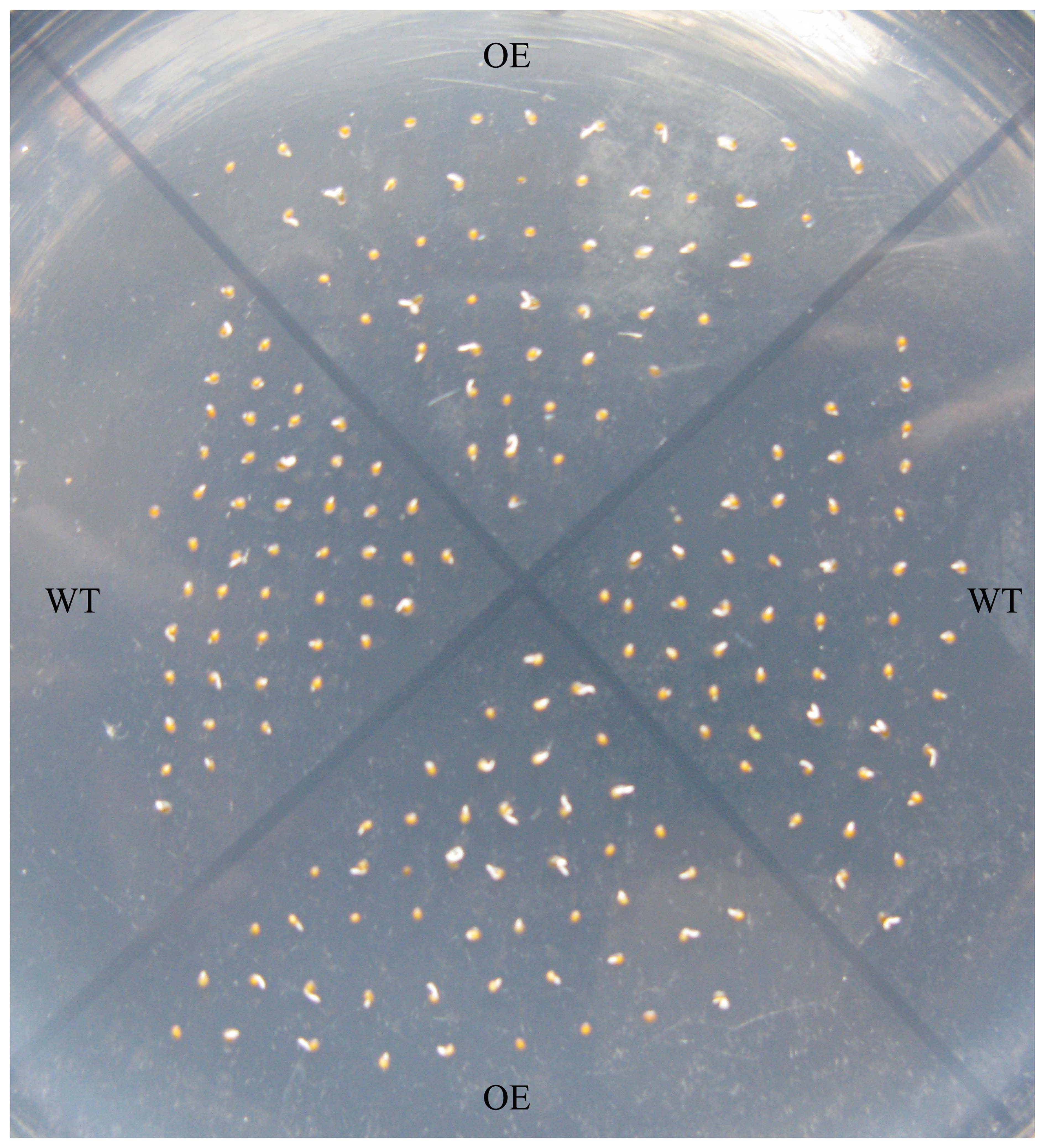

Supplement: FIGURE S3 — Germination status of transgenic Arabidopsis seeds after 7 days on a culture dish. The seeds of SaCu/Zn SOD transgenic (OE2, OE3, and OE4) and WT plants were surface-sterilized and plated on 1/2 MS agar medium plates supplemented with 1.5 mM CdCl2 and grown 7 days. [file Image_3.JPEG]

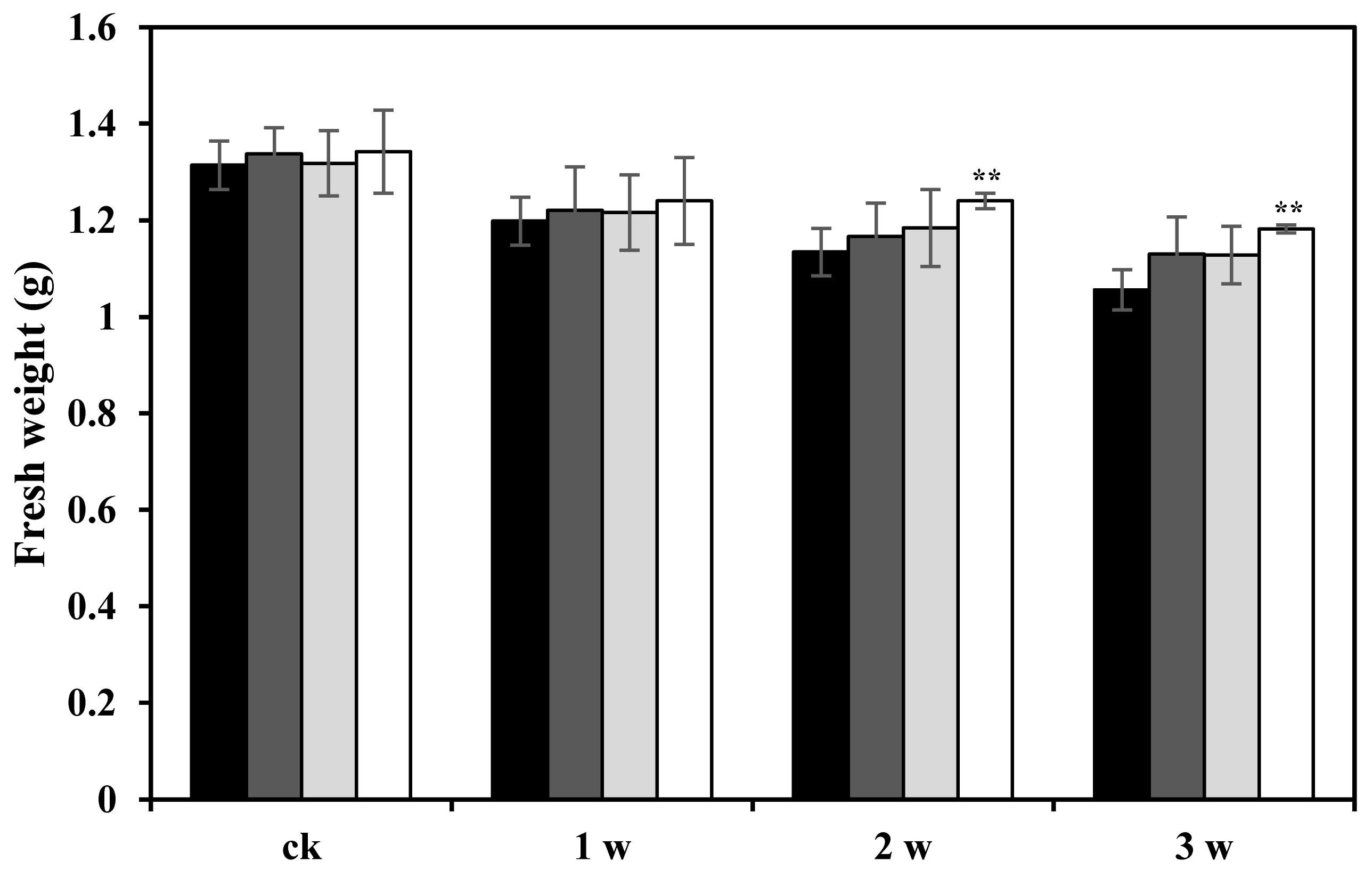

Supplement: FIGURE S4 — Fresh weights of transgenic and WT Arabidopsis exposed to Cd for different time periods. Bars indicate means ± SD. Asterisks indicate significant differences at ∗p < 0.05 and ∗∗p < 0.01. [file Image_4.JPEG]

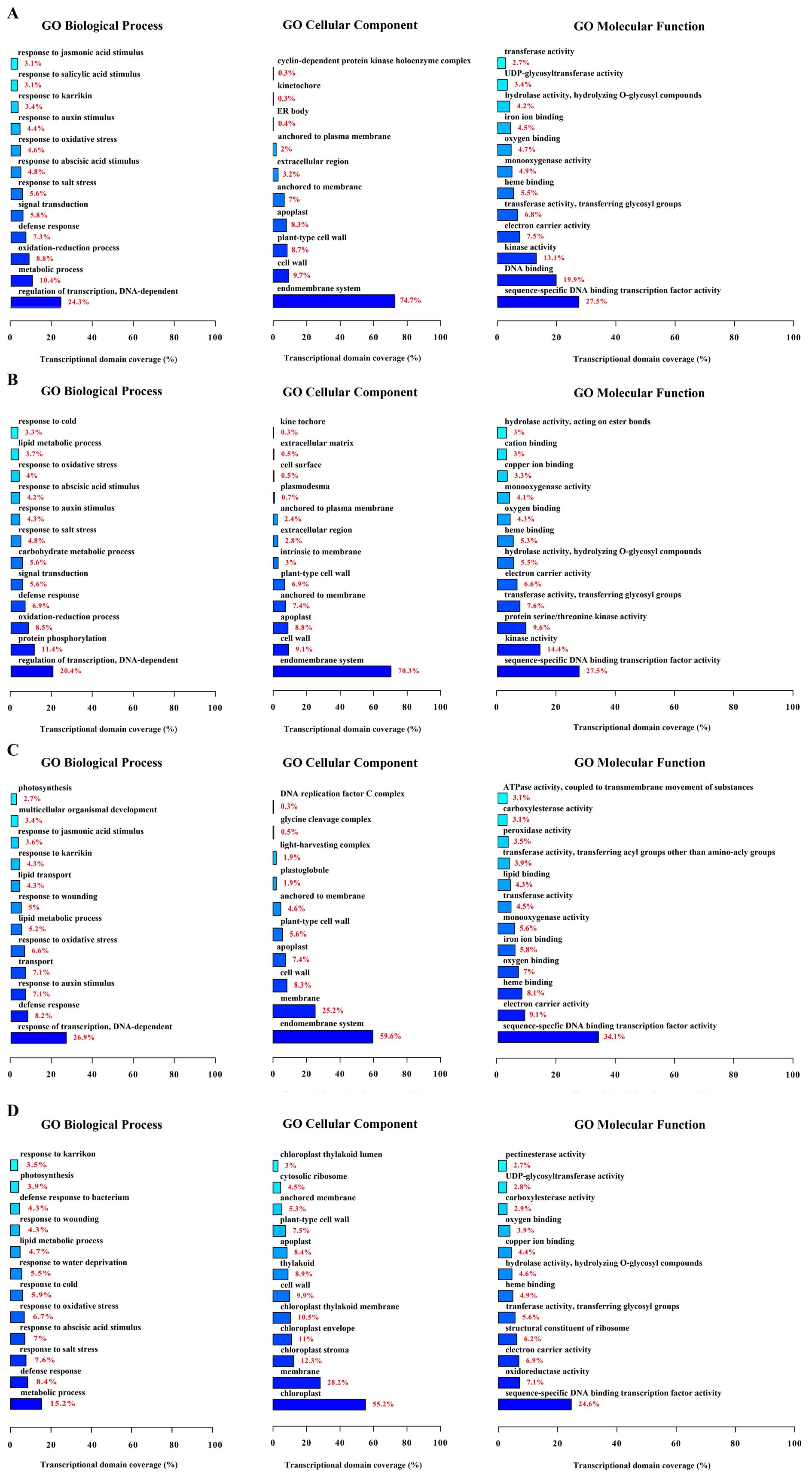

Supplement: FIGURE S5 — Gene ontology analyses of the differentially regulated genes identified by microarray analysis under normal growth and Cd stress conditions. (A–D) indicate 0 week (ck), 1 week, 2 weeks, and 3 weeks, respectively. GO analysis of each period includes “Biological process,” “Cellular component,” and “Molecular function”. [file Image_5.JPEG]
