# Supplementary material for: Overexpressing the Sedum alfredii Cu/Zn Superoxide Dismutase Increased Resistance to Oxidative Stress in Transgenic Arabidopsis
Source: Front Plant Sci. 2017 Jun 13;8:1010. doi: 10.3389/fpls.2017.01010 (PMC5469215; doi:10.3389/fpls.2017.01010)
Supplement: TABLE S1 — Primers used in this study. [file Table_1.DOCX]

**Table S1 List of primers**

| **Names** | **Sequences (5'→3')** | **Purpose** |
| --- | --- | --- |
| SOD-F | ATGGCGGCTCAGACTGTCAT | cDNA/DNA amplication |
| SOD-R | TTAAGTGGGTGTCAGCCCTATC |  |
| RT-F | GCTGCATCTCGACAGGACCA | RT-PCR |
| RT-R | CTACAAACGCACGCCCAACA |  |
| UCE9-F | TGGCGTCGAAAAGGATTCTGA |  |
| UCE9-R | CCTTCGGTGGCTTGAATGGAT |  |
| TUB-F | TTATGGCGATTCCGAGCTTCA |  |
| TUB-R | ATTATTTCCAGCGCCGGATTG |  |
| ACT2-F | TTCCGGTGATGGTGTCAGTCA |  |
| ACT2-R | ACAATTTCCCGCTCAGCAGTG |  |
| Actin-F | GCACCCTGTTCTTCTTACCG |  |
| Actin-R | AACCCTCGTAGATTGGCACA |  |
| α-tubulin-F | GATGTACCGTGGTGATGTC |  |
| α-tubulin-R | GAGCCTCTGAAAATTCTCC |  |
| Ubiquitin-F | GGAAAGCAGCTCGAAGATG |  |
| Ubiquitin-R | AAGCTTCCACCGCGGAGAC |  |
| NPT-F | ATCTCCTGTCATCTCACCTTGCTCCT | trangenic plants selection |
| NPT-R | TCAGAAGAACTCGTCAAGAAG |  |
